# Evidence of Circadian Rhythm, Oxygen Regulation Capacity, Metabolic Repeatability and Positive Correlations between Forced and Spontaneous Maximal Metabolic Rates in Lake Sturgeon *Acipenser fulvescens*

**DOI:** 10.1371/journal.pone.0094693

**Published:** 2014-04-09

**Authors:** Jon C. Svendsen, Janet Genz, W. Gary Anderson, Jennifer A. Stol, Douglas A. Watkinson, Eva C. Enders

**Affiliations:** 1 Environmental Science, Fisheries and Oceans Canada, Winnipeg, Manitoba, Canada; 2 Interdisciplinary Centre of Marine and Environmental Research, University of Porto, Porto, Portugal; 3 Biology Department, University of West Georgia, Carrollton, Georgia, United States of America; 4 Department of Biological Sciences, University of Manitoba, Winnipeg, Manitoba, Canada; The Evergreen State College, United States of America

## Abstract

Animal metabolic rate is variable and may be affected by endogenous and exogenous factors, but such relationships remain poorly understood in many primitive fishes, including members of the family Acipenseridae (sturgeons). Using juvenile lake sturgeon (*Acipenser fulvescens*), the objective of this study was to test four hypotheses: 1) *A. fulvescens* exhibits a circadian rhythm influencing metabolic rate and behaviour; 2) *A. fulvescens* has the capacity to regulate metabolic rate when exposed to environmental hypoxia; 3) measurements of forced maximum metabolic rate (MMR_F_) are repeatable in individual fish; and 4) MMR_F_ correlates positively with spontaneous maximum metabolic rate (MMR_S_). Metabolic rates were measured using intermittent flow respirometry, and a standard chase protocol was employed to elicit MMR_F_. Trials lasting 24 h were used to measure standard metabolic rate (SMR) and MMR_S_. Repeatability and correlations between MMR_F_ and MMR_S_ were analyzed using residual body mass corrected values. Results revealed that *A. fulvescens* exhibit a circadian rhythm in metabolic rate, with metabolism peaking at dawn. SMR was unaffected by hypoxia (30% air saturation (O_2sat_)), demonstrating oxygen regulation. In contrast, MMR_F_ was affected by hypoxia and decreased across the range from 100% O_2sat_ to 70% O_2sat_. MMR_F_ was repeatable in individual fish, and MMR_F_ correlated positively with MMR_S_, but the relationships between MMR_F_ and MMR_S_ were only revealed in fish exposed to hypoxia or 24 h constant light (i.e. environmental stressor). Our study provides evidence that the physiology of *A. fulvescens* is influenced by a circadian rhythm and suggests that *A. fulvescens* is an oxygen regulator, like most teleost fish. Finally, metabolic repeatability and positive correlations between MMR_F_ and MMR_S_ support the conjecture that MMR_F_ represents a measure of organism performance that could be a target of natural selection.

## Introduction

Animal metabolic rate is variable and may be influenced by both endogenous factors (e.g. circadian rhythm, individual physiological traits) and exogenous factors (e.g. oxygen availability). A surge of research interest continues to uncover the mechanistic basis of variability in metabolic rate [1], and metabolic rate is now one of the most widely measured physiological traits in animals [2]. In many aquatic animals, measurements of oxygen consumption rate (*M*O_2_) provide a robust proxy for aerobic metabolic rates. Under static conditions, measurements of *M*O_2_ are typically repeatable in individual animals, suggesting that metabolic rate may be an organismal trait [3], although the repeatability tends to decline over time [2].

Circadian rhythms in physiology and behaviour have evolved to allow animals to anticipate changes in the light-dark environment that are tied to the rotation of Earth. Circadian rhythms reflect endogenous rhythms that are self-sustained, unlike exogenous rhythms that depend on external factors, including changing light levels [4]. Circadian rhythms play a tremendous role in most organisms; ranging from decentralized regulation of the daily timing of mitosis [5] to influencing the migration of animals [6]. Circadian rhythms have been described in details in several teleost fishes [4], [5], [7], [8]. For example, circadian rhythms influencing metabolic rate and behaviour have been documented in Nile tilapia *Oreochromis niloticus* [9] and puffer fish *Takifugu obscurus* [10]. In contrast, in many primitive fishes, the influence of circadian rhythms on metabolism and behaviour remains largely unknown.

Standard metabolic rate (SMR) is a basic maintenance requirement measured as the minimum rate of oxygen consumption of postprandial unstressed animals at rest [11]. Long-term energy demands for swimming, food acquisition and treatment, regulation owing to environmental perturbation, and reproduction are additional to standard metabolism [11]. These demands are met within the range set by the maximum metabolic rate (MMR) [11].

Animal metabolic physiology is often influenced by exogenous factors, including environmental hypoxia. Hypoxia occurs in a wide range of aquatic systems [12], and the severity, frequency of occurrence, and spatial scale of hypoxia have increased in the last few decades, primarily due to anthropogenic activity [13], [14]. There are two distinct metabolic responses to environmental hypoxia: 1) oxygen independent respiration in which the metabolic rate remains constant in spite of changing oxygen availability; and 2) oxygen dependent respiration in which the metabolic rate varies with oxygen availability [15]. The two responses are commonly termed oxygen regulation and oxygen conformity, respectively. The vast majority of literature suggests that most teleost fish are oxygen regulators [16]–[20], capable of maintaining both MMR and SMR down to certain oxygen thresholds [21], [22]. In contrast, it remains controversial if oxygen regulation or conformity occurs in a number of primitive fishes exposed to hypoxia. For example, among members of the family Acipenseridae (sturgeons), previous studies have reported conflicting results stating that the metabolic rate remains constant or tends to increase [23]–[26] (i.e. oxygen regulator) or decrease [27]–[30] (i.e. oxygen conformer) when Acipenserids are exposed to environmental hypoxia. Using Adriatic sturgeon *Acipenser naccarii*, McKenzie et al. [31] suggested that swimming *A. naccarii* are oxygen regulators, whereas immobile *A. naccarii* are oxygen conformers. Knowing whether species are oxygen regulators or conformers is important to understand the capacity of fish to respond to environmental changes [20] and to assess assumptions for disparate metabolic theories in ecology [19].

Intraspecific variation in animal metabolic rate may correlate with endogenous factors, including behavioural or life history traits [32], [33]. For example, Niitepõld and Hanski [34] found positive correlations between MMR and life span in a species of butterfly. In fish, MMR is typically measured in the laboratory using either a critical swimming protocol [35] or a chase protocol [36]. Using the latter protocol, Norin and Malte [3] reported that MMR is repeatable over several weeks. Assuming repeatability and heritability, MMR may represent a measure of organism performance [3], and it is possible that the trait is subjected to natural selection and could evolve over time. Little is known, however, about potential correlations between forced MMR (MMR_F_; e.g. measured using the chase protocol) and spontaneous MMR (MMR_S_) measured in volitionally performing fish. For example, is there a positive relationship between MMR_F_ and MMR_S_ such that an individual fish with an unexpectedly high MMR_F_ also has an unexpectedly high MMR_S_? Clarifying potential correlations between MMR_F_ and MMR_S_ is important, because from an evolutionary point of view, selection regimes may not always operate on a trait's maximal value, but rather on the spontaneous use of the trait [37], [38]. If MMR_F_ and MMR_S_ are correlated, measurements of MMR_F_ could function as a predictor of MMR_S_ in individual fish.

Using juvenile lake sturgeon (*Acipenser fulvescens*), we employed intermittent flow respirometry and video analysis to test four hypotheses: 1) *A. fulvescens* exhibit a circadian rhythm influencing metabolic rate and behavior; 2) *A. fulvescens* has the capacity to regulate metabolic rate when exposed to environmental hypoxia; 3) measurements of MMR_F_ are repeatable in individual fish, and 4) MMR_F_ is positively correlated with MMR_S_.

Our results reveal that the metabolic rate of *A. fulvescens* is influenced by a circadian rhythm, and *A. fulvescens* has the capacity to regulate SMR when exposed to environmental hypoxia, demonstrating oxygen regulation. In contrast, MMR_F_ tends to decrease with increasing levels of hypoxia. Measurements of residual body mass corrected MMR_F_ are repeatable in individual *A. fulvescens*; and residual body mass corrected MMR_F_ and MMR_S_ are correlated positively, but only in *A. fulvescens* exposed to an environmental stressor including hypoxia or 24 h of light.

## Materials and Methods

### Ethics statement

All procedures were reviewed and approved by the Animal Care Committee at the University of Manitoba, Canada (Approval ID: AUP-F11-004) under the guidelines of the Canadian Council of Animal Care. No animals were sacrificed, all efforts were taken to ameliorate animal suffering and undue stress, and there was no mortality during any of the tests.

### Experimental animals

A total of 70 juvenile *A. fulvescens* (body mass: 30.51±1.21 g (mean ± S.E.); age: 1+; sex: unknown) obtained from Grand Rapids Fish Hatchery (Grand Rapids, MB, Canada) were kept at 17±1°C in flow-through holding tanks at the University of Manitoba, Canada. The light regime was 12 h light: 12 h dark (12L∶12D). *A. fulvescens* were fed daily using a mixture of bloodworm (San Francisco Bay Brand, Newark, CA, USA) and sinking trout pellet (Martin Mills Ltd., Elmira, ON, Canada).

### Respirometry

Four static respirometers (each 0.83 l) and a mixing pump were submerged in a 100 l opaque tank, filled with freshwater maintained at 17±0.1°C. Oxygen content (% air saturation; O_2sat_) of the water in the tank was controlled using two air stones combined with a stream of nitrogen bubbles [39]. Depending on the experiment, water in the tank was maintained at an oxygenation level between 100% and 30% O_2sat_.

Respirometers were made of transparent glass tubing and were designed to allow a degree of spontaneous activity of *A. fulvescens*, including body undulations with tail excursions>90° relative to the body axis. Respirometers were situated in a sound isolated room with no other ongoing experiments to minimize any disturbance of the fish.

Measurements of *M*O_2_ (mg O_2_ h^−1^) were carried out every 9 min using computerized intermittent flow respirometry allowing long term (>48 h) repeated measurements [40]. Each respirometer was fitted with two outlet and two inlet ports as described previously [41]. The repeated respirometric loops consisted of a 4 min flushing phase during which a pump flushed the respirometer with ambient water through one set of ports. The second set of ports and a pump secured re-circulation of water in the respirometer in a closed circuit phase for 5 min, divided into a waiting phase (2 min) and a measurement phase (3 min).

Oxygen partial pressure was measured at 1 Hz by a fiber optic sensor (Fibox 3 connected to a dipping probe; PreSens, Regensburg, Germany) located in the re-circulated loop. The flush pump was controlled by AutoResp software (version 2.1.3; Loligo Systems, Tjele, Denmark) that also calculated the *M*O_2_ in the measurement phase using the oxygen partial pressure and standard equations [42], [43]. Preliminary testing demonstrated that the duration of the measurement phase (3 min) ensured that the coefficient of determination (r^2^) associated with each *M*O_2_ measurement was always>0.95, similar to previous studies [44]. Corrections of background respiration (i.e. microbial respiration) followed Jones et al. [45].

### Experimental protocols

*A. fulvescens* were selected randomly and fasted for 48 h to ensure a post absorptive state prior to experimentation. Subsequently, *A. fulvescens* were introduced to the respirometers and acclimated for 20 h. The light regime during the fasting and acclimation periods was 12L∶12D, which included 0.5 h of gradually shifting light intensity from light to darkness and *vice versa*. Light intensities were 3.0 and 0.0 μmol s^−1^ m^−2^ in daylight and darkness, respectively. Starting at 16:00 h on the next day, *M*O_2_ data were collected for the following 24 h.

Measurements of *M*O_2_ over 24 h comprised three test groups: 1) control (100% O_2sat_; 12L∶12D); 2) treatment A (30% O_2sat_; 12L∶12D); and 3) treatment B (100% O_2sat_; 24L). The oxygen content in treatment A (30% O_2sat_) corresponded to approximately 6.2 kPa. Data collection for the three test groups was carried out in a random fashion and each test group included 10–12 individuals. After each 24 h trial, MMR_F_ was measured as described below.

### Standard metabolic rate (SMR) and maximum metabolic rates (MMR_F_ and MMR_S_)

For each test group, SMR in individual fish was estimated as the average of the lowest 10 *M*O_2_ values collected over 24 h. This method to estimate SMR was employed because it provides measurements that are repeatable in individual fish [3].

MMR_F_ was measured immediately after each 24 h trial at the corresponding O_2sat_ level (i.e. 100% or 30% O_2sat_) inside the respirometer. MMR_F_ was elicited using a standard chase protocol [36]. Briefly, individual *A. fulvescens* were transferred to a circular trough and chased to exhaustion, similar to previous studies on Atlantic sturgeon (*Acipenser oxyrhynchus*) and shortnose sturgeon (*Acipenser brevirostrum*) [46]. Upon exhaustion, identified by no further response after 5 min of manual stimulation, *A. fulvescens* were transferred (<20 s) to the respirometer where *M*O_2_ measurements started immediately. MMR_F_ was the highest of three consecutive *M*O_2_ measurements.

In addition, following the same chase protocol, MMR_F_ was measured in 36 *A. fulvescens* exposed to 100%, 90%, 80% or 70% O_2sat_ inside the respirometer. A total of 8–12 *A. fulvescens* were tested at each of the four O_2sat_ levels. Measurements of MMR_F_ in 100% O_2sat_ were repeated after 4.5 h to examine the short term repeatability of MMR_F_ in individual fish. These two measurements were termed initial and final MMR_F_.

Finally, for each test group (i.e. control and treatments A and B), MMR_S_ was estimated as the single highest measurement of *M*O_2_ (i.e. one respirometric loop) in volitionally performing individual fish during the complete 24 h trial (i.e. after acclimation). These data were used to test for correlations between MMR_F_ and MMR_S_ in individual fish (see Data analysis).

### Behaviour

*A. fulvescens* in the respirometers were recorded (25 frames s^−1^) dorsally using a UEye camera (model UI-1640SE-C-GL; IDS, Woburn, MA, USA) equipped with a CCTV lens (model HF6M-2; Spacecom, Whittier, CA, USA). The software UEye Cockpit (version 3.90; IDS, Woburn, MA, USA) was used to download recordings to a PC. Two Scene illuminators (model S8030-30-C-IR; Guangdong, China) provided infra-red light for nocturnal recordings. All recordings were synchronized with the respirometric loops (to the nearest 1 s). For each *A. fulvescens*, behavioural data were collected over a 45 s time interval during the measurement phase of the respirometric loop (i.e. once every 9 min.). Behavioural data included total activity (i.e. % of time moving), and the number of body undulations with tail excursions<90° or >90° relative to the body axis (i.e. body undulations min^−1^). For each test group, behavioural data were collected over a 1 h time interval (i.e. 6–7 respirometric loops) at 16, 20, 21, 22 and 23 h. These hourly measurements were selected to record simultaneous metabolic and behavioural changes during the light-dark transition at 21 h.

### Data analysis

*M*O_2_ data were body mass adjusted following previous studies [47]. Metabolic rates from the three test groups were calculated over 1 h intervals [48], with two exceptions, because the light intensity was gradually changing over 0.5 h periods at 21 h and 9 h. Therefore, the two 1 h intervals associated with 21 h and 9 h were each divided into two: 0.5 h with changing light intensities and 0.5 h with constant light intensity. The compiled data were used to compare metabolic rates over 24 h within the three test groups (i.e. control and treatments A and B). Behavioural data were compiled in the same fashion.

Metabolic and behavioural variables were compared within each test group across the time interval from 16:00 to 23:00 h using a repeated measure (RM) one way ANOVA. Relationships between behaviour and metabolic rates were investigated using least squares linear regression.

To test for metabolic differences, SMR, MMR_F_ and MMR_S_ measurements were compared between the three test groups using a one way ANOVA. MMR_F_ data from the four oxygen treatments (100 – 70% O_2sat_) were analyzed using least square linear regression to examine the effect of decreasing oxygen levels on MMR_F_.

The method recommended by Norin and Malte [3] was used to examine repeatability of the MMR_F_ measurements. All values of MMR_F_ and body mass were log_10_-transformed prior to the analysis. Mass-independent data of MMR_F_ were expressed as residual values using the relationship between body mass and MMR_F_. Fish with higher than expected MMR_F_ have positive residuals and fish with lower than expected MMR_F_ have negative residuals. Repeatability of the two sets of residuals (initial and final) was estimated using Spearman's rank correlation coefficient (ρ) [3].

Using metabolic rate data from the three test groups, MMR_S_ of each individual fish was extracted to test for correlations between individual MMR_F_ and MMR_S_. The comparison of individual MMR_F_ and MMR_S_ was carried out in the same fashion as the repeatability analysis described above.

Log_10_ transformations of data prior to statistical analysis were employed to meet assumptions of normal distribution of data and homogeneity of variance. If the assumptions were met, ANOVA or RM ANOVA were employed depending on design as described above. If significant, the tests were followed by pairwise multiple comparisons using the Holm-Sidak method.

If data transformations did not permit the use of parametric testing, ANOVA on ranks or RM ANOVA on ranks (Friedman) were employed depending on design as described above. The tests were followed by pairwise multiple comparisons using Dunn's method to take unequal sample sizes into account.

Tests were carried out using SigmaStat 3.01 (Systat Software, San Jose, CA, USA) and SPSS 20.0 (IBM, Armonk, NY, USA). Results were considered significant if α<0.05. All values are reported as means ± S.E. unless noted otherwise.

## Results

For all the experiments, there were no indications that the health status of the test animals changed during any of the tests.

### Body mass adjustments

**Figure 1:**
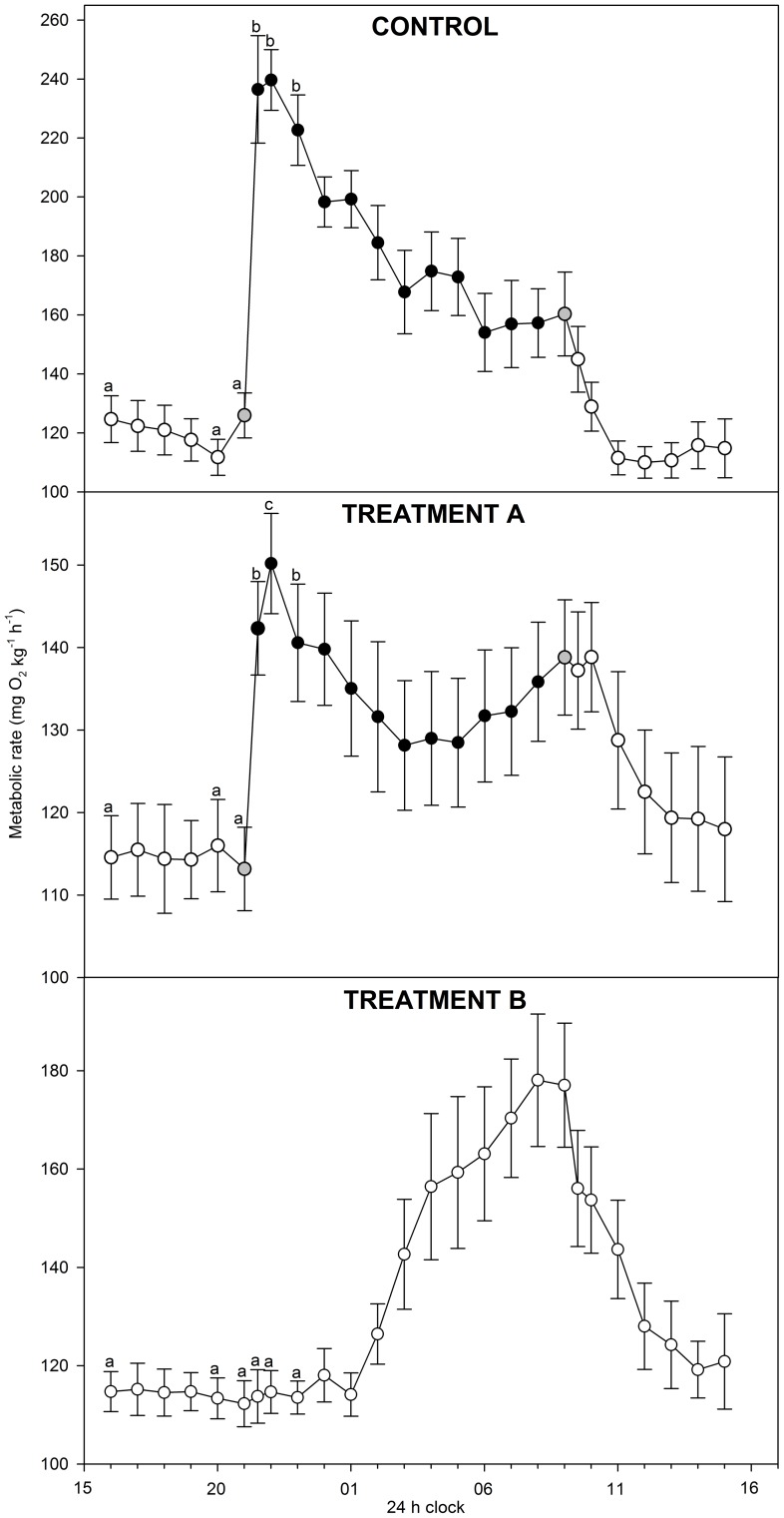
Metabolic rates (mg O_2_ kg^−1^ h^−1^) over 24 h in lake sturgeon *Acipenser fulvescens*. Data collection comprised three test groups: control (100% O_2sat_; 12L∶12D), treatment A (30% O_2sat_; 12L∶12D), and treatment B (100% O_2sat_; 24L). Colours of the symbols indicate light levels with white, black and grey data points representing light, dark and intermediate light levels, respectively. Different letters indicate significant (P<0.05) differences between measurements within each test group. Note that y-axes differ between the three panels.

There were no differences between test groups (i.e. control, treatments A and B) in terms of body mass and SMR measured as mg O_2_ h^−1^ (both P>0.05). Consequently, SMR data were pooled, and the relationship between log_10_ SMR and log_10_ body mass was described using a linear equation [3], [47]. The slope of the relationship was 1.00±0.12 indicating that a 1.0 body mass scaling coefficient was appropriate for the SMR data. A 1.0 body mass scaling coefficient is consistent with two previous studies on green sturgeon *Acipenser medirostris* [47], [49]. Because the 1.0 body mass scaling coefficient was appropriate for the SMR data, the same coefficient was used for the *M*O_2_ data collected over time (Fig. 1).

MMR_F_ measured as mg O_2_ h^−1^ did not differ between the control group and treatment B (P>0.05), but MMR_F_ from treatment A was lower than both the control group and treatment B (P<0.001). To examine the relationship between body mass and MMR_F_ (mg O_2_ h^−1^), data collected in normoxia were combined and the relationship between log_10_ MMR_F_ and log_10_ body mass was described using a linear equation [3], [47]. The slope of the relationship was 0.90±0.05 indicating that a 0.9 body mass scaling coefficient was appropriate for the MMR_F_ data. Consequently, all MMR_F_ data were standardized to the mean body mass of 30.5 g using 0.9 as the body mass scaling coefficient. In the following, MMR_F_ standardized to 30.5 g is denoted MMR_F30.5_.

### Metabolic rates over 24 h

Metabolic rates varied substantially over the 24 h periods (Fig. 1). In the control group, metabolic rate increased significantly (P<0.001) from 112 mg O_2_ kg^−1^ h^−1^ at 20:00 h to reach a maximum of 237 mg O_2_ kg^−1^ h^−1^ when the light went off (Fig. 1, control), indicating a dusk metabolic peak. Thereafter, metabolic rate decreased and reached 157 mg O_2_ kg^−1^ h^−1^ shortly before daylight. The metabolic rate decreased further in daylight and reached 112 mg O_2_ kg^−1^ h^−1^ after 3 h.

In treatment A, metabolic rate increased significantly (P<0.05) from 116 to 150 mg O_2_ kg^−1^ h^−1^ when the light went off (Fig. 1, treatment A). Although truncated, this metabolic peak corresponded to the dusk metabolic peak observed in the control test group. Thereafter, metabolic rate decreased to 128 mg O_2_ kg^−1^ h^−1^ at 03:00 h, and then increased to reach 139 mg O_2_ kg^−1^ h^−1^ during the period with increasing light intensity (09:00 h). Thus, treatment A indicated two metabolic peaks; one associated with dusk and one associated with dawn. After the light went on, the metabolic rate changed little for 1 h and then decreased to 118 mg O_2_ kg^−1^ h^−1^ (Fig. 1, treatment A).

In treatment B, the metabolic rate remained below 119 mg O_2_ kg^−1^ h^−1^ until 02:00 h (Fig. 1, treatment B). Data showed that the dusk metabolic peak, observed in the control group and in treatment A, was eliminated by the constant light (P = 0.64). In contrast, in treatment B, the metabolic rate tended to increase at 02:00 h and continued doing so until it reached 178 mg O_2_ kg^−1^ h^−1^ at 08:00 h (Fig. 1, treatment B). These data indicated the presence of a darkness independent increase in the metabolic rate. The increasing metabolic rate peaked around dawn, just before the light would normally come on.

Collectively, data indicated the presence of two metabolic peaks occurring over 24 h. The first metabolic peak occurred around dusk and was noticeable in the control group and treatment A (Fig. 1). The second metabolic peak occurred around dawn and was noticeable in treatments A and B (Fig. 1).

### Behaviour across the light-dark transition

**Figure 2:**
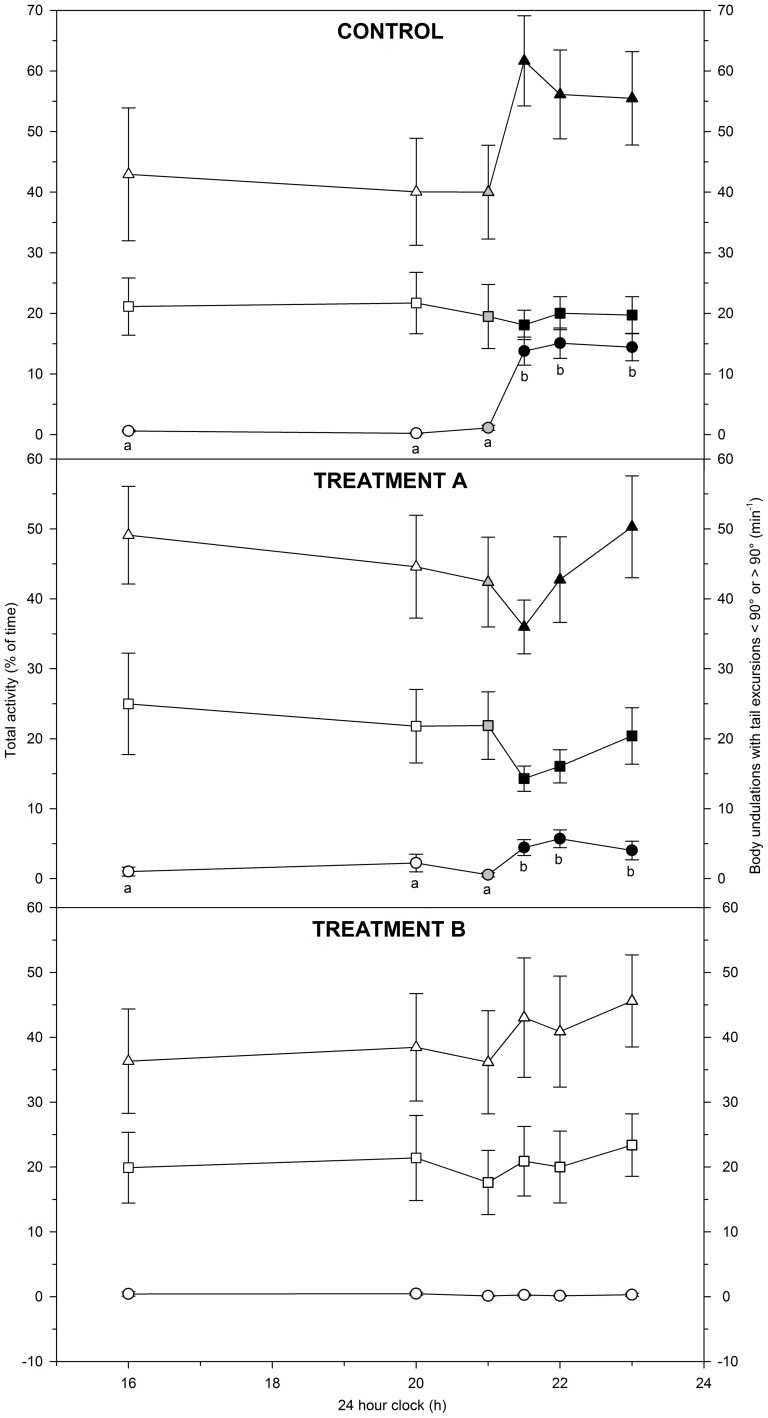
Hourly behavioural variables in lake sturgeon *Acipenser fulvescens* from 16:00 h to 23:00 h. Data collection comprised three test groups: control (100% O_2sat_; 12L∶12D), treatment A (30% O_2sat_; 12L∶12D), and treatment B (100% O_2sat_; 24L). Colours of the symbols indicate light levels with white, black and grey data points representing light, dark and intermediate light levels, respectively. Behavioural variables included total activity (% of time moving) (triangles) and the frequencies of body undulations with tail excursions<90° (squares) or >90° (circles) (min^−1^). Within each test group, behavioural variables were compared over time to identify significant changes. Different letters indicate significant (P<0.05) changes over time, whereas identical or no letters indicate non-significant (P>0.05) changes over time.

Behavioural recordings from the control group indicated that the total activity increased in darkness (Fig. 2, control), but no statistically significant differences were identified over time (P>0.05). Similarly, the frequency of body undulations with tail excursions<90° did not change significantly over time (P>0.05). In contrast, body undulations with tail excursions>90° increased significantly over time (P<0.001) (Fig. 2, control).

Data from treatment A revealed no significant changes in the total activity over time or in the frequency of body undulations with tail excursions<90° (both P>0.05) (Fig. 2, treatment A). In contrast, the frequency of body undulations with tail excursions>90° increased significantly over time (P<0.001).

Data from treatment B revealed no significant changes over time in the total activity or in the frequencies of body undulations with tail excursions<90° or >90° (all P>0.05; Fig. 2, treatment B).

### Correlations between behaviour and metabolic rate

**Figure 3:**
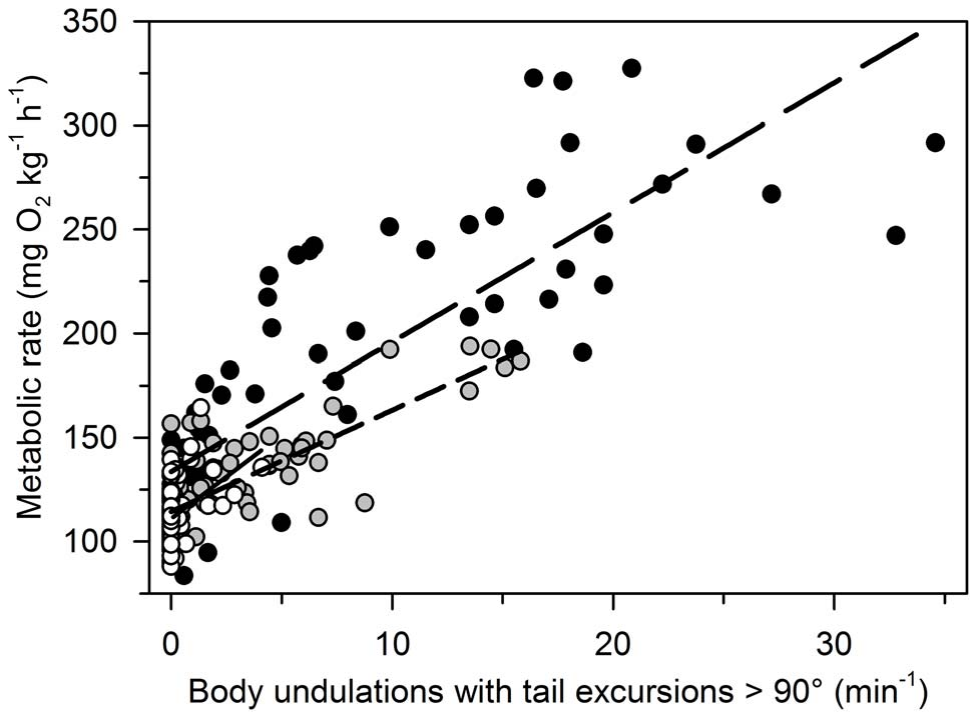
Metabolic rates (mg O_2_ kg^−1^ h^−1^) correlate positively with behaviour in lake sturgeon *Acipenser fulvescens*. Behaviour involved body undulations with tail excursions>90° (min^−1^). Data were collected from 16:00 h to 23:00 h. Data collection comprised three test groups: control (100% O_2sat_; 12L∶12D), treatment A (30% O_2sat_; 12L∶12D), and treatment B (100% O_2sat_; 24L). Note that symbol colours indicate the three test groups: control (black symbols; long dash line), treatment A (gray symbols; short dash line) and treatment B (white symbols; solid line). The three corresponding linear least squares regressions are highly significant (all P<0.001) and the coefficients of determination (r^2^) are 0.68, 0.64 and 0.15, respectively.

The behavioural data suggested that the frequency of body undulations with tail excursions>90° (Fig. 2) could be a major driver of the increase in metabolic rate associated with dusk (Fig. 1). Regression analysis revealed highly significant (P<0.001 in all cases) linear relationships between the frequency of body undulations with tail excursions>90° and metabolic rate (Fig. 3). The coefficients of determination (r^2^) for the relationships varied between test groups and were 0.68, 0.64 and 0.15 for control and treatments A and B, respectively (Fig. 3). These data suggest that metabolic variation was coupled with behavioural variation.

### Environmental effects on standard metabolic rate (SMR) and forced maximum metabolic rate (MMR_F_)

SMR was unaffected by hypoxia (treatment A) and constant light (treatment B) (P>0.05; Table 1), and the pooled average was 92.39±2.00 mg O_2_ kg^−1^ h^−1^. Corresponding analyses of MMR_F30.5_ revealed no differences between the control and treatment B (P>0.05), whereas MMR_F30.5_ from treatment A was lower than both the control and treatment B (P<0.001; Table 1). These findings showed that 30% O_2sat_ reduced MMR_F30.5_.

**Table 1:** Metabolic variables (mean ± S. E.) in lake sturgeon *Acipenser fulvescens* representing three different test groups: control (100% O_2sat_; 12L∶12D); treatment A (30% O_2sat_; 12L∶12D); and treatment B (100% O_2sat_; 24L).

Metabolic variable	Control	Treatment A	Treatment B
SMR (mg O_2_ kg^−1^ h^−1^)	88.44±3.54^a^	97.23±4.06^a^	91.50±2.34^a^
MMR_F30.5_ (mg O_2_ kg^−1^ h^−1^)	338.25±8.06^a^	167.49±5.81^b^	328.43±8.29^a^
MMR_S_ (mg O_2_ kg^−1^ h^−1^)	311.91±13.60^a^	168.72±7.57^b^	265.24±18.44^c^

Sample size (n) is 8–12 for each test group. Different superscript letters indicate significant differences (P<0.05) between test groups. SMR is the standard metabolic rate. MMR_F30.5_ and MMR_S_ are the forced and spontaneous maximum metabolic rates, respectively. Measurements of MMR_F30.9_ are body mass adjusted to a 30.5 g fish. Body mass adjustments of MMR_S_ to a 30.5 g fish (i.e. equivalent to MMR_F30.5_) change MMR_S_ values by <1% and have no impact on the conclusions.

**Figure 4:**
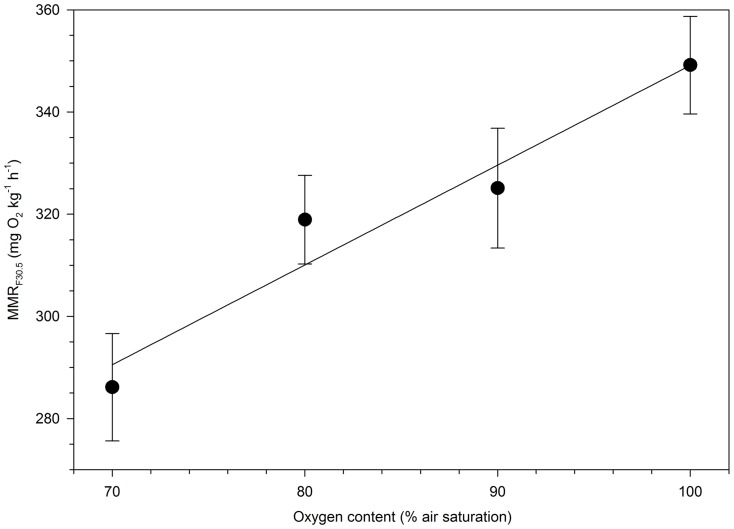
Forced maximum metabolic rate (MMR_F30.5_) is influenced by hypoxia in lake sturgeon *Acipenser fulvescens*. Measurements of MMR_F30.5_ are body mass adjusted to a 30.5 g fish. MMR_F30.5_ decreased significantly across the range from 100% O_2sat_ to 70% O_2sat_ (P<0.03; r^2^>0.94).

MMR_F30.5_ was quantified in four separate groups of *A. fulvescens* exposed to 100%, 90%, 80% or 70% O_2sat_ to estimate the effect of hypoxia on MMR_F30.5_. Body mass did not differ between the four treatments (P = 0.95). MMR_F30.5_ was affected by hypoxia and decreased across the range from 100% O_2sat_ to 70% O_2sat_ (Fig. 4) as revealed by the linear regression analysis (P<0.03; r^2^>0.94). These findings indicated that the maximum metabolic rate of *A. fulvescens* is sensitive to increasing levels of hypoxia.

### Repeatability of forced maximum metabolic rates (MMR_F_)

**Figure 5:**
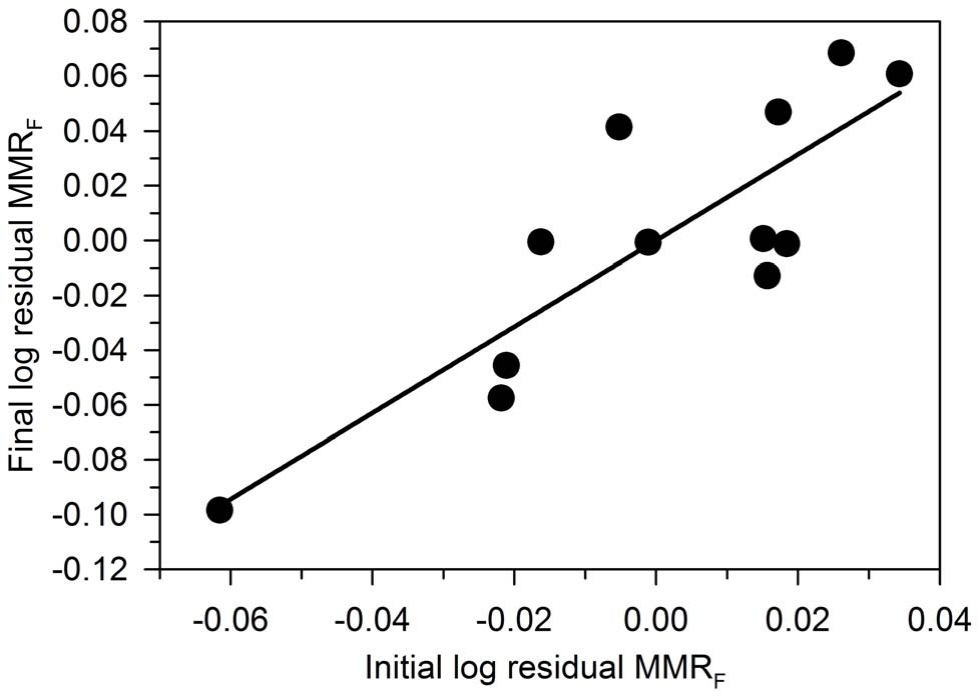
Forced maximum metabolic rate (MMR_F_) is repeatable in individual lake sturgeon *Acipenser fulvescens*. Spearman's rank statistics were used to test for correlations between initial and final residual (i.e. body mass corrected) maximum metabolic rate (residual MMR_F_; mg O_2_ h^−1^) measured in individual *A. fulvescens*. The significant relationship (P<0.006; ρ = 0.76) indicates repeatability of MMR_F_. Time interval between initial and final measurements was 4.50 h.

Analysis of repeatability followed a previous study [3] and showed that measurements of residual body mass corrected MMR_F_ are repeatable in individual fish. Spearman's rank correlation coefficient (ρ) for the relationship between the initial and final residual MMR_F_ was 0.76, and the relationship was highly significant (P<0.006) (Fig. 5).

### Spontaneous maximum metabolic rate (MMR_S_)

MMR_S_ was extracted from each 24 h trial for comparisons between test groups. MMR_S_ differed significantly between all three test groups (P<0.05) (Table 1). These findings showed that MMR_S_ was suppressed in treatments A and B, with the most pronounced effect in treatment A (Table 1). Standardizing MMR_S_ to a 30.5 g fish using a 0.9 body mass scaling coefficient (i.e. equivalent to MMR_F30.5_) changed MMR_S_ values by < 1% and had no impact on the conclusions.

### Correlations between forced (MMR_F_) and spontaneous (MMR_S_) maximum metabolic rates

**Figure 6:**
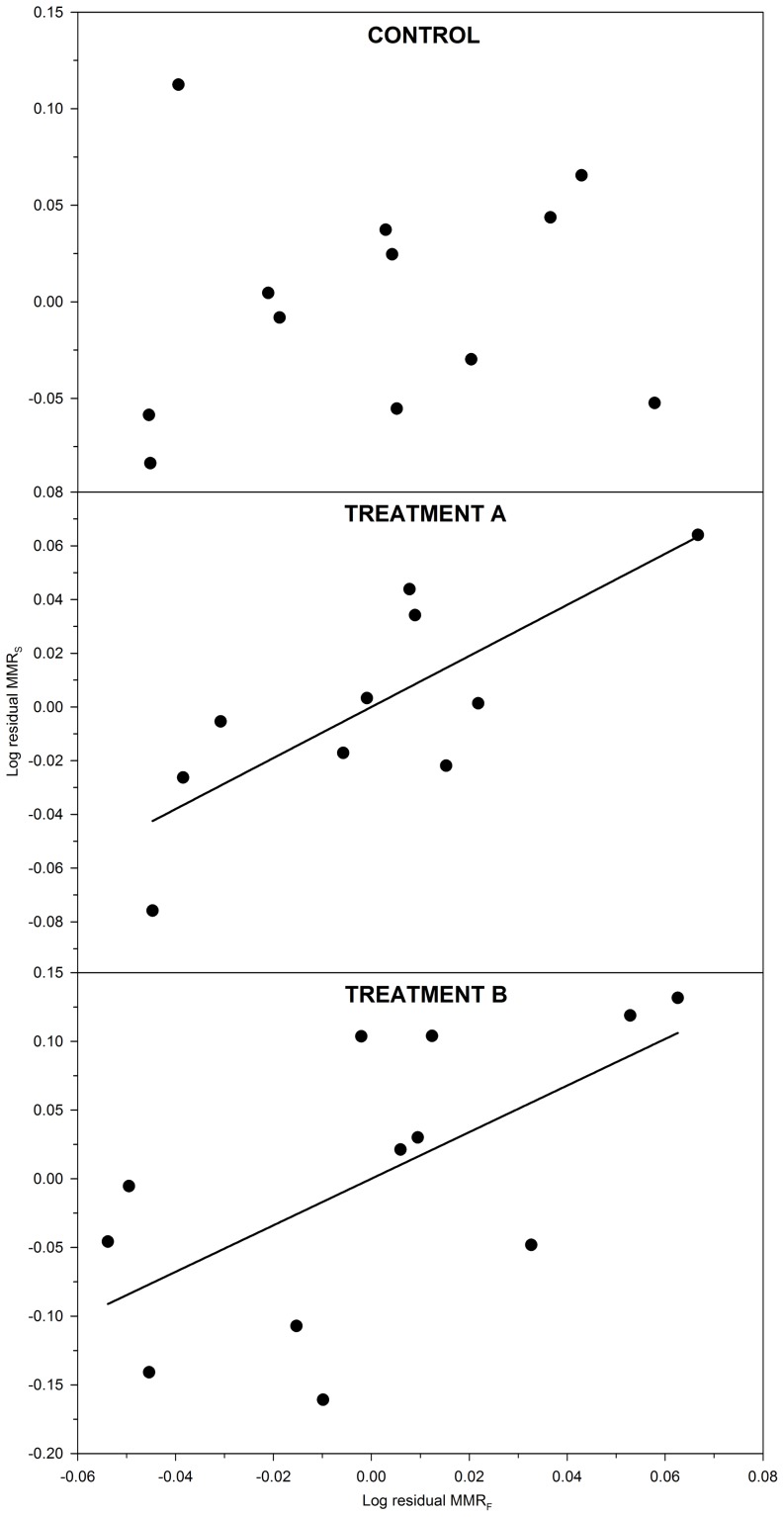
Relationships between forced and spontaneous maximum metabolic rates in lake sturgeon *Acipenser fulvescens*. Data collection comprised three test groups: control (100% O_2sat_; 12L∶12D), treatment A (30% O_2sat_; 12L∶12D), and treatment B (100% O_2sat_; 24L). Spearman's rank statistics were used to test for correlations between forced (MMR_F_) and spontaneous (MMR_S_) residual (i.e. body mass corrected) maximum metabolic rate (mg O_2_ h^−1^) measured in individual *A. fulvescens*. In the control group, there was no significant relationship between the residuals (P = 0.40; ρ = 0.27). In contrast, the residuals correlated positively in both treatments A and B (both P<0.05; ρ≥0.66).

MMR_F_ and MMR_S_ were compared to test the hypothesis that they would correlate positively. Data showed that residual MMR_F_ and residual MMR_S_ were not correlated in the control group (P = 0.40; ρ = 0.27) (Fig. 6, control). In contrast, residual MMR_F_ and residual MMR_S_ were positively correlated in both treatments A (P<0.05; ρ = 0.69) and B (P<0.05; ρ = 0.66) (Fig. 6, treatments A and B). These data indicated that an individual with an unexpectedly high MMR_F_ also has an unexpectedly high MMR_S_, at least when the individual is exposed to an environmental stressor, such as hypoxia (treatment A) or constant light (treatment B).

## Discussion

This study provides evidence that the organismal physiology of *A. fulvescens* is influenced by a circadian rhythm and strongly indicates that *A. fulvescens* is an oxygen regulator. Using residual (i.e. body mass corrected) values, the study suggests that MMR_F_ is repeatable in individual *A. fulvescens*, and MMR_F_ can be positively correlated with MMR_S_. The relationship between MMR_F_ and MMR_S_ appears, however, to depend on the presence of an environmental stressor such as hypoxia or constant light.

Our data indicated the presence of two metabolic peaks in *A. fulvescens* occurring over 24 h (Fig. 1). The first metabolic peak occurred around dusk (control group and treatment A), whereas the second metabolic peak occurred around dawn (treatments A and B) (Fig. 1). The dusk metabolic peak was eliminated by the constant light in treatment B, suggesting that the dusk metabolic peak reflected an exogenous rhythm, depending on exogenous stimuli (i.e. decreasing light levels). In contrast, the dawn metabolic peak occurred regardless of constant light, suggesting that a circadian rhythm, including an endogenous mechanistic basis, control the metabolic rate of *A.* fulvescens. As far as is known, our study provides the first evidence of a circadian rhythm in Acipenserids. It is not clear why the dawn metabolic peak was not distinct in the control group (Fig. 1). We suggest that the relatively high metabolic rates masked the dawn metabolic peak in the control group. In the hypoxic treatment, metabolic rates were suppressed, but not to an extent where the dawn metabolic peak was eliminated (Fig. 1). Therefore, the metabolic suppression in hypoxia helped revealing the underlying presence of two metabolic peaks.

In a recent field study, Forsythe et al. [50] reported that adult *A. fulvescens* initiate upstream migration around dusk and dawn. The authors suggested that the observations could ultimately be explained by reduced risk of predation and harvest by humans at dusk and dawn [50]. While the present study used juvenile *A. fulvescens*, our data indicate that the migratory peaks at dusk and dawn observed by Forsythe et al. [50] could reflect proximate mechanisms that include an exogenous rhythm at dusk and a circadian rhythm at dawn.

This study tested the hypothesis that *A. fulvescens* is an oxygen regulator. Our data provide two lines of evidence that *A. fulvescens* is an oxygen regulator, capable of regulating metabolic rate and maintaining metabolic rhythms in environmental hypoxia. Firstly, we found no evidence that SMR differed between normoxia and hypoxia (30% O_2sat_) (Table 1). Thus, *A. fulvescens* maintained SMR regardless of fluctuating environmental oxygen levels. Secondly, *A. fulvescens* exposed to hypoxia (30% O_2sat_) exhibited a similar metabolic rate rhythm over the time interval from 16 h to 23 h as *A. fulvescens* exposed to normoxia and was capable of increasing the metabolic rate around dusk in the hypoxic environment (Fig. 1, treatment A). The metabolic increase had a strong behavioural component in both hypoxia and normoxia, and correlated positively with the frequency of body undulations with tail excursions>90° (Fig. 3). These data show that *A. fulvescens* is capable of regulating metabolic rate (SMR) and maintaining metabolic rhythms in hypoxia. Thus, *A. fulvescens* is an oxygen regulator, like most teleost fishes.

In contrast to SMR, data indicated that MMR_F30.5_ is sensitive to increasing levels of hypoxia in *A. fulvescens* (Fig. 4). Physiologically, the result is expected because if a fish is exercising at MMR before the hypoxic exposure, compensatory mechanisms (e.g. increasing gill ventilation and cardiac output) are already utilized to support the elevated oxygen requirements and are unavailable to compensate for environmental hypoxia. The result is not, however, consistent with previous studies on teleost fish. Most previous studies have reported that the maximum metabolic rate in normoxia is maintained in low levels of hypoxia [21], [22], [41], typically down to approximately 80% O_2sat_. The reason for the discrepancy between the present and previous studies remains unknown, but is it possible the maximum metabolic rate of *A. fulvescens* is more sensitive to low levels of hypoxia than in most teleost fishes. Further tests comparing Acipenserids and teleost fishes using identical equipment and experimental approaches are required to examine the discrepancy.

Previous studies have demonstrated that SMR and MMR are repeatable physiological traits in a wide range of taxa [2]. Repeatability (or temporal consistency) is important when ascribing certain properties to an individual animal on the basis of a single physiological measurement [3]. Repeatability indexes the reliability of the protocol used to measure a trait [51] and further sets a general upper limit to the intensity of selection that can be applied to the trait [52]. If a trait is not repeatable over time, a single measure of the trait may not be representative of future physiological performance and it becomes unlikely that natural selection can act on the trait, i.e. separate the favoured from disfavoured individuals [53]. Little is known about repeatability of traits in Acipenserids, but a recent behavioural study [54] demonstrated that spawning times and locations are highly repeatable in mature *A. fulvescens*. To our knowledge, the present study provides the first estimate of physiological repeatability in Acipenserids. Our data suggest that body mass corrected measurements of MMR_F_ are repeatable in *A. fulvescens* (Fig. 5), at least over short time intervals (4.5 h) and set the stage for studies examining repeatability over longer time intervals.

Recently, it has been shown that not only SMR and MMR, but also routine metabolic rate (RMR) can be a repeatable trait in fish [55]. Repeatability of RMR suggests that the spontaneous activity within a respirometer is not simply random bouts of movement over time, but rather, that individual fish exhibit consistent behavioural patterns when evaluated at different times [55]. The present study tested whether body mass corrected values of MMR_F_ and MMR_S_ are positively correlated to examine whether an unexpectedly high value of MMR_F_ would indicate an unexpectedly high value of MMR_S_. By demonstrating positive relationships between MMR_F_ and MMR_S_ in *A. fulvescens* exposed to an environmental stressor (Fig. 6), the present study adds to the growing body of evidence indicating that variation in metabolism, as determined over time in a respirometer, is not random, but may reflect physiological or behavioural traits in individual animals.

Measurements of physiological performance, including MMR_F_ and critical swimming speed (*U*_crit_), are widely used whole-organism indicators of maximal performance, examined to better understand evolutionary and physiological ecology [3], [53], [56]–[61]. While maximal performance is crucial for a wide range of behaviours tightly connected to fitness (e.g. [62], [63]), animals may not exercise at maximal intensity very often [64]–[66]. Therefore, measurements of maximal performance could have more pronounced functional importance if maximal performance correlated positively with spontaneous performance, which is used more frequently. In particular, this is important because selection regimes may not only operate on a trait's maximal value, but alternatively on the spontaneous use of the trait (i.e. ecological performance [37], [38]). In the present study, we examined maximal forced and spontaneous performances by measuring MMR_F_ and MMR_S_ to test whether the two traits are correlated. Considering treatments A and B, data indicated that *A. fulvescens* exhibiting an unexpectedly high MMR_F_ also exhibit an unexpectedly high MMR_S_ (Fig. 6). These data suggest that MMR_F_ may be indicative of MMR_S_ in individual *A. fulvescens*. Nevertheless, we only found relationships between MMR_F_ and MMR_S_ when fish were exposed to an environmental stressor (hypoxia or 24 h light), and no relationship when fish were exposed to normoxia and a normal light regime (12L∶12D) (Fig. 6).

It remains unclear why we observed relationships between MMR_F_ and MMR_S_ when *A. fulvescens* were exposed to an environmental stressor, and no relationship without an environmental stressor (Fig. 6). Our findings are, however, consistent with a recent review by Killen et al. [1]. The authors described how environmental stressors, including hypoxia and light, may either reveal or mask relationships between behaviour and physiology. Because we found evidence of correlations between behaviour and metabolic rate (Fig. 3), it is likely that MMR_S_ not only reflected a physiological trait, but also a behavioural trait. As such, our relationships between MMR_F_ and MMR_S_ (Fig. 6) could be considered relationships between physiology and behaviour that were revealed by environmental stressors, as suggested by Killen et al. [1]. All our measurements of MMR_F_ were stressful for *A. fulvescens* [46], [67], whereas the measurements of MMR_S_ were probably most stressful under hypoxia and constant light. Physiological stress is associated with increased concentrations of plasma cortisol in Acipenserids [67]–[69] with secondary responses involving metabolism [70]. In the present study, MMR_S_ was suppressed in treatments A and B (Table 1), and stress experienced by *A. fulvescens* under hypoxia and constant light could have influenced the relative distribution of phenotypes with regard to MMR_S_, such that positive correlations between MMR_F_ and MMR_S_ were revealed in treatments A and B (see Fig. 1 in Killen et al. [1]). This remains speculation, however, and further studies of the coupling between behaviour and physiology in divergent environments are needed to evaluate the hypothesis.
